# Angioinvasive *Trichophyton rubrum* associated necrotizing fasciitis in an immunocompromised patient

**DOI:** 10.1002/ccr3.7718

**Published:** 2023-09-30

**Authors:** Michael J. Davis, Katelyn J. Rypka, Alexandra K. Perron, John Keilty, Benjamin Wils, Joshua Levine, Anthony T. Rezcallah, Robin Solomon, Noah Goldfarb, Anjum Kaka

**Affiliations:** ^1^ Minneapolis VA Health Care System – Department of Infectious Diseases and University of Minnesota – Department of Infectious Diseases Minneapolis Minnesota USA; ^2^ Minneapolis VA Health Care System – Department of Dermatology and University of Minnesota – Department of Dermatology Minneapolis Minnesota USA; ^3^ University of Minnesota Medical School Minneapolis Minnesota USA; ^4^ Minneapolis VA Health Care System – Department of Surgery and University of Minnesota – Department of Surgery Minneapolis Minnesota USA; ^5^ Department of Pathology Minneapolis VA Health Care System Minneapolis Minnesota USA; ^6^ Department of Infectious Diseases Minneapolis VA Health Care System Minneapolis Minnesota USA

**Keywords:** angioinvasive dermatophytosis, hematologic malignancy, immunosuppression, necrotizing fasciitis, terbinafine, Trichophyton rubrum

## Abstract

Angioinvasive dermatophytosis with necrotizing fasciitis can be a rare complication in immunocompromised patients with early surgical debridement, 12 weeks of oral terbinafine, and reduction in immunosuppression being a viable management strategy.

## Graphical abstract

*Trichophyton rubrum* hyphae demonstrated within a blood vessel from debrided lower extremity tissue (Grocott's methenamine silver stain, magnification 20x).

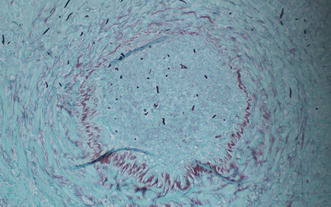

## 1 INTRODUCTION

Dermatophyte infections are characterized by local invasion of superficial keratinized structures such as skin, hair, and nails by members of the genera *Trichophyton*, *Microsporum*, and *Epidermophyton*.^1^, ^2^ Organisms from these genera infect keratinocytes via arthrospores released from hyphae. Following attachment to keratinocytes, these arthrospores germinate, and establish infection in the stratum corneum of the skin.^2^ These infections clinically manifest as annular plaques with a collarette of scale. In humans, these infections are limited to the superficial layers of the skin due to the keratinophilic nature of the organism as well as intact cell mediated immunity limiting spread of the infection to deeper tissues.^1^, ^3^, ^4^ Uncommonly, when cell mediated immunity is impaired, these organisms can invade down the hair follicle resulting in Majocchi granuloma. Rarely deeper invasion may extend into the dermis and subcutaneous tissue.^5^, ^6^ With an increasingly large variety of immunosuppressive medications on the market to treat patients with autoimmune conditions, malignancies, and solid organ transplants, there is an increasing population of immunosuppressed patients who may be at risk for invasive dermatophyte infections. This case presents a patient with recurrent tinea corporis who developed angioinvasive dermatophytosis in the setting of ongoing immunosuppressive treatment for chronic lymphocytic leukemia (CLL) and immune thrombocytopenia purpura (ITP).

## 2 CASE PRESENTATION

A 69‐year‐old male with a history CLL, ITP, and recurrent tinea corporis presented to the hospital with an ulcerating lesion at the site of a previously diagnosed tinea corporis infection of his right medial ankle.

**FIGURE 1:**
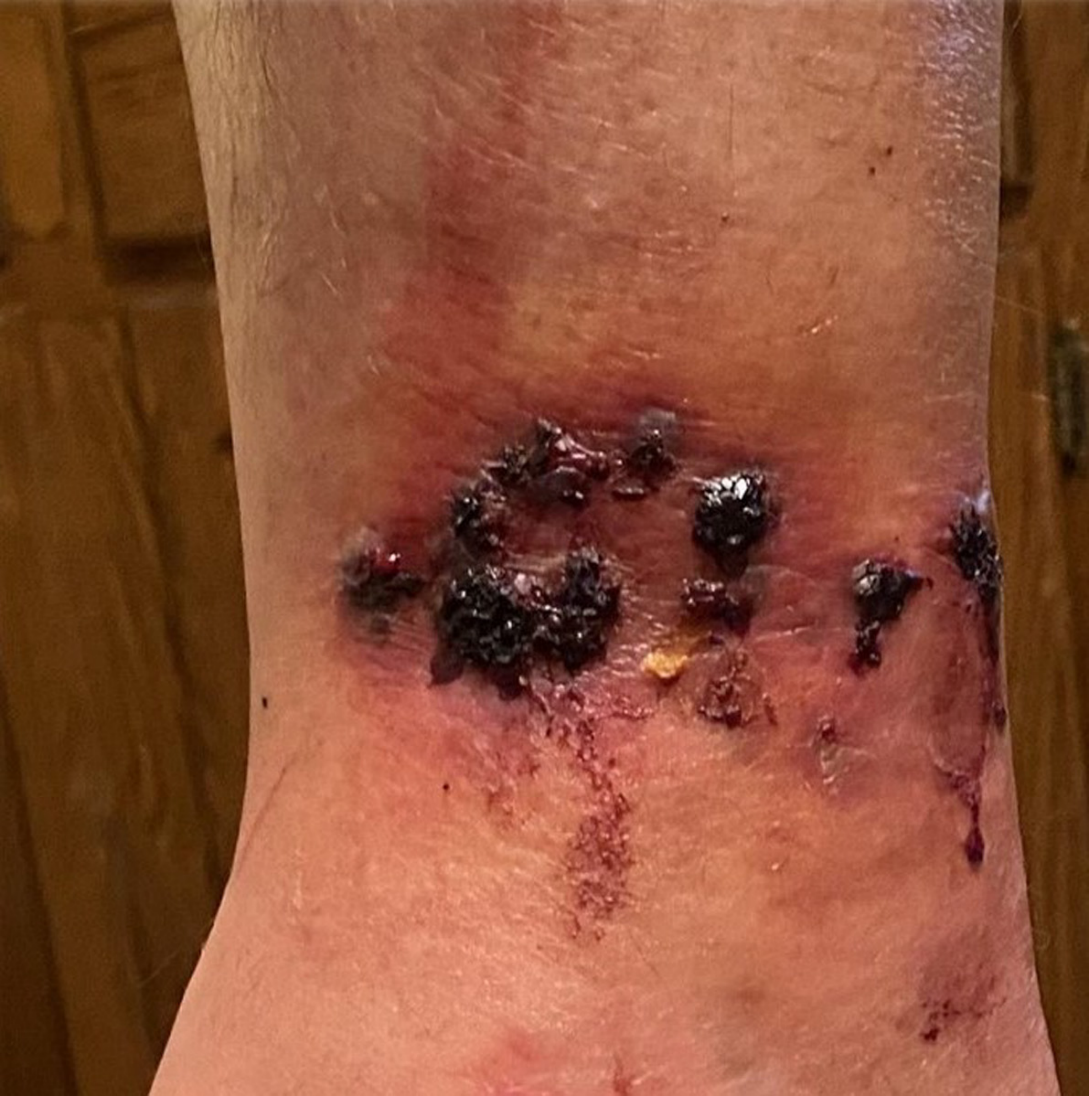
Well‐demarcated annular plaque of the medial right lower extremity with dusky hemorrhagic crusts.

One month prior to presentation, the patient was started on high‐dose steroids for treatment of refractory ITP. After 2–3 weeks of experiencing a limited response to steroid treatment, he was started on rituximab and ultimately hospitalized for refractory ITP. During this hospitalization, the patient's ITP was thought to be triggered by CLL prompting the initiation of ibrutinib for treatment. Following the initiation of ibrutinib, a new pruritic, painful skin lesion on his right medial ankle developed which was evaluated by dermatology. This skin lesion was painful to the touch and noted to be a well‐demarcated bright red plaque with scattered hemorrhagic, dusky, purple papules within the plaque. There was a collarette of scale overlying the edge of the lesion. A skin scraping with potassium hydroxide preparation demonstrated numerous branching hyphae. A biopsy of the lesion was deferred due to thrombocytopenia (11,000/cmm). Topical terbinafine 1% cream twice daily was started for presumed superficial tinea corporis infection. The patient was subsequently discharged once his thrombocytopenia stabilized. Less than 1 week from discharge, the patient's distal right lower extremity became progressively more edematous with new ulceration and serosanguinous drainage (Figure 1). He presented to the hospital again and was admitted for a second time (Table 1). Further evaluation with a computed tomography scan of the right lower extremity demonstrated diffuse subcutaneous edema with pooling of fluid along the superficial fascia. There were no signs of focal fluid collection or subcutaneous emphysema. Due to clinical concern for necrotizing fasciitis, the patient was urgently taken to the operating room by general surgery where he underwent four‐compartment fasciotomy of the right lower extremity as well as excisional debridement of the involved area at the right ankle until healthy appearing and bleeding tissue was reached.

**FIGURE 2:**
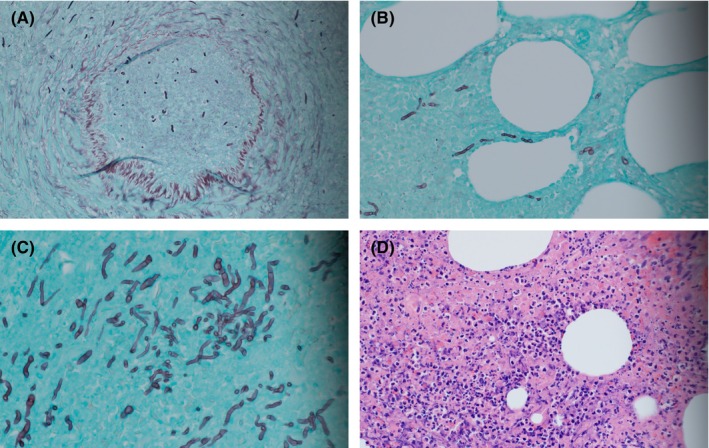
(A) Fungal elements demonstrated within a blood vessel from debrided right lower extremity tissue (Grocott's methenamine silver stain, magnification 20x). (B) Septated fungal elements demonstrated within subcutaneous tissue (Grocott's methenamine silver stain, magnification 40x). (C) Tissue demonstrating branching, septated fungal elements (Grocott's methenamine silver stain, magnification 60x). (D) Subcutaneous tissue demonstrating purulent inflammation (Hematoxylin and Eosin stain, magnification 40x).

**TABLE 1:** Events preceding hospital admission for dermatophyte associated necrotizing fasciitis.

Day	Event
1	Prednisone 50 mg BID, 14‐day course
15	Dexamethasone 40 mg daily, 4‐day course
22	Rituximab infusion
24	Hospital admission for refractory thrombocytopenia
25	IVIG infusion Prednisone 50 mg BID started Ibrutinib 420 mg daily started
26	IVIG infusion
29	Rituximab infusion Right lower extremity lesion noted Prednisone dose change from 50 mg BID to 80 mg daily
30	Dermatology consulted KOH of skin scraping with branching fungal hyphae Topical terbinafine 1% cream BID application
31	Discharge from hospital
32	Prednisone dose change from 80 mg daily to 60 mg daily
36	Hospital admission for necrotizing fasciitis of right lower extremity

Intraoperatively, there was extensive necrosis involving the dermis, subcutaneous adipose tissue, and superficial fascial layers which were debrided. The underlying muscle layers, tendons, and deep fascia appeared alive and healthy. Tissue was sent for culture and pathology. At the fasciotomy sites in the leg, there was a positive finger sign between the adipose and the fascia as well as between the fascia and the muscle, without frankly necrotic tissue in this part of the leg. While the fascia and muscle appeared healthy, this clinical finding of easy separation between the two tissue planes was thought to be representative of early‐stage infection in which necrosis of the epimysium occurred without progression to widespread tissue necrosis. Therefore, no further sharp debridement was performed. Pulse lavage irrigation and debridement with nine liters of normal saline was then performed at the surgical sites.

**FIGURE 3:**
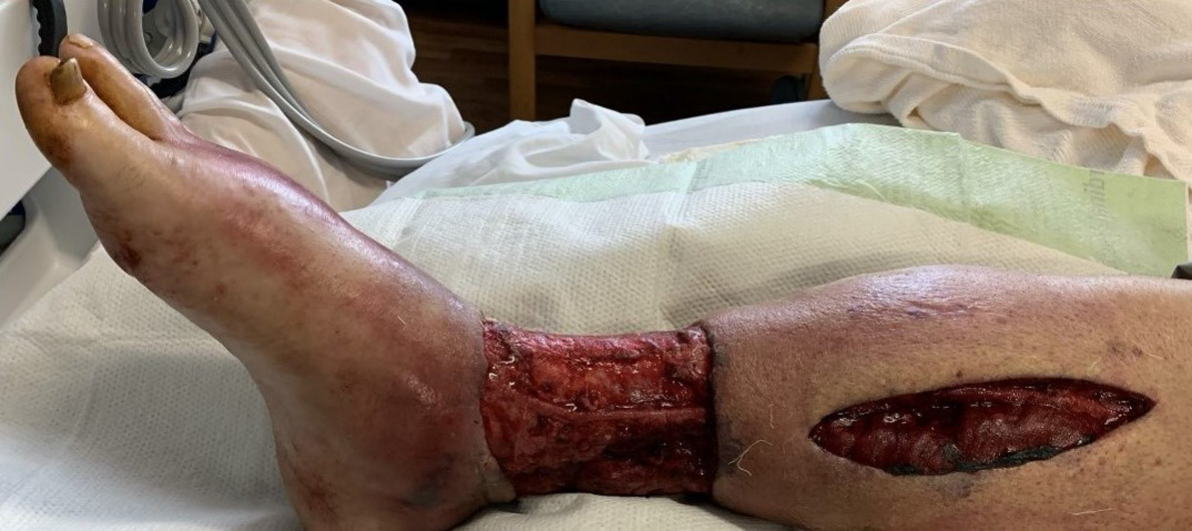
Right lower extremity wound postoperative day 6 after four compartment fasciotomy and debridement.

Operating room cultures grew *Trichophyton rubrum*, as well as methicillin‐susceptible *Staphylococcus aureu*s and *Enterococcus faecalis*. Pathology exam demonstrated numerous branching, septated fungal hyphae within purulent inflammation in subcutaneous tissue, fascia, and also intravascularly within a thrombosed blood vessel (Figure 2). On postoperative day three, the patient developed elevated temperatures and was noted to have skin findings on his left lower extremity similar to the early stages of his right lower extremity infection. On postoperative day four, he underwent excisional debridement of his new left lower extremity lesion at bedside. Deep tissue specimens of his left lower extremity lesion grew *T. rubrum* and pathological exam demonstrated purulent inflammation with necrosis and branching septated fungal hyphae. On postoperative day six, his right lower extremity wounds showed stability (Figure 3).

Ultimately, this patient received daily wound care with multiple dressing changes which allowed for his surgical wounds to granulate and fill in appropriately. He was treated with a three‐week course of antibacterials for methicillin susceptible *S. aureus* and *E. faecalis* cultured from the right lower extremity, and oral terbinafine 250 mg daily for 12 weeks for his invasive dermatophyte infection of both extremities. Given the extensive debridement, his right lower extremity wounds were covered with split thickness skin grafts 6 weeks postoperatively. At a clinic visit 2 weeks after completing terbinafine treatment, the patient had well healed skin grafts with no signs of recurrent infection.

## 3 DISCUSSION

Given the keratinophilic nature of dermatophytes, invasion deeper than the stratum corneum in the skin is rare.^1^ However, this case demonstrates that increased immunosuppression in patients with superficial dermatophyte infections may predispose to angioinvasive disease involving deeper tissues. The most common predisposing factors associated with invasive dermatophyte infections include: superficial dermatophytosis, solid organ transplant, topical immunosuppressants, gene mutations, diabetes, and trauma.^6^ The development of our patient's angioinvasive dermatophyte infection was preceded by increasing immunosuppression with high dose steroids, rituximab, and ibrutinib in the setting of worsening CLL and ITP. It is known that immunosuppressive agents used in hematologic malignancies such as bruton tyrosine kinase inhibitors (ibrutinib) and anti‐CD20 monoclonal antibodies (rituximab) are associated with an increased risk of invasive fungal infection.^7^, ^8^, ^9^, ^10^ Classically, these fungal infections include invasive yeast infections such as candidiasis, mold infections including aspergillosis and fusariosis, endemic fungal disease, and classic opportunistic infections such as cryptococcus and pneumocystis jiroveci.^8^ Invasive dermatophyte infections are rarely considered when increasing a patient's immunosuppression. In the setting of superficial dermatophyte infections, clinicians should be aware of the possibility of invasive disease when increasing immunosuppression similar to the more classic non‐dermatophyte invasive fungal infections. When a superficial dermatophyte infection presents with a dusky appearance and/or leads to pain out of proportion to exam, there should be concern for invasive disease, including necrotizing fasciitis, and early biopsy/surgical intervention should be considered.

Antifungal treatment guidance for invasive dermatophyte infections is scarce. When dermatophyte infections extend beyond the stratum corneum, it is generally recommended to transition from topical to oral therapy. There is not a consensus on the preferred oral antifungal. A recent systematic review of invasive dermatophyte infections showed that the most commonly used agents were terbinafine and itraconazole.^6^ However, a number of patients were treated with other antifungals including griseofulvin, fluconazole, amphotericin B, and posaconazole.^6^ There was additional variability in the duration of treatment for the cases reviewed. We treated our patient with oral terbinafine 250 mg daily for 12 weeks. This drug, dose, and duration of therapy in combination with surgical debridement and reduced immunosuppression resulted in clinical clearance of infection.

## 4 CONCLUSIONS

This case demonstrates a rare presentation of an angioinvasive dermatophyte necrotizing soft tissue infection in the setting of hematologic malignancy and increasing immunosuppression. In patients with superficial dermatophyte infections, immunosuppression may predispose to deeper infection and potentially angioinvasive disease. If superficial dermatophyte lesions appear dusky with pain out of proportion to exam, early biopsy should be pursued to assess for deeper invasion. Early and aggressive surgical debridement with assessment and washout of the fascial compartments can halt progression of necrotizing soft tissue infections and result in limb salvage. This case additionally adds to the paucity of literature on management strategies of angioinvasive dermatophyte infections which included surgical debridement, reduction of immunosuppression, and a 12‐week course of oral terbinafine 250 mg daily.

## AUTHOR CONTRIBUTIONS

**Michael J. Davis:** Conceptualization; writing – original draft; writing – review and editing. **Katelyn J. Rypka:** Conceptualization; writing – original draft; writing – review and editing. **Alexandra K. Perron:** Writing – review and editing. **John Keilty:** Writing – review and editing. **Benjamin Wils:** Writing – review and editing. **Joshua Marc Levine:** Writing – review and editing. **Anthony T. Rezcallah:** Supervision; writing – review and editing. **Robin Solomon:** Resources. **Noah Goldfarb:** Supervision; writing – review and editing. **Anjum Kaka:** Supervision; writing – review and editing.

## CONFLICT OF INTEREST STATEMENT

Dr. Goldfarb has participated in clinical trials with Abbvie, Pfizer, Chemocentrix and DeepX Health, and has served on advisory boards and consulted for Novartis and Boehringer Ingelheim. The content of this article is solely the responsibility of the authors and does not necessarily represent the official views of any other companies or organizations.

## CONSENT

Written informed consent was obtained from the patient to publish this report in accordance with the journal's patient consent policy.

## Data Availability

Data sharing not applicable ‐ no new data generated
